# Effect of Stabilization Treatment on the Microstructural Evolution and Tensile Properties of GH4706 Superalloy

**DOI:** 10.3390/ma18184297

**Published:** 2025-09-13

**Authors:** Jialiang Huang, Ran Duan, Xiangyi Hou, Chong Wang, Xintong Lian, Shuo Huang

**Affiliations:** 1 School of Materials Science and Engineering, Shanghai University, Shanghai 200444, China; shuhjl@shu.edu.cn (J.H.); houxiangyi@shu.edu.cn (X.H.); 2 Gaona Aero Material Co., Ltd., Beijing 100081, China; duanran@cisri-gaona.com.cn (R.D.); huangshuohank@163.com (S.H.); 3 The Key Laboratory for Anisotropy and Texture of Materials (Ministry of Education), School of Materials Science and Engineering, Northeastern University, Shenyang 110819, China; 2190025@stu.neu.edu.cn

**Keywords:** nickel-based superalloy, stabilization treatment, tensile properties, microstructural evolution, elemental segregation

## Abstract

GH4706 Ni-based superalloy is widely used for aero-engine turbine disks operating below 700 °C, where high-temperature ductility is critical to avoid cracking during die forging and service. However, the microscopic mechanisms by which stabilization treatment regulates its high-temperature ductility remain insufficiently clarified. This study systematically investigated the tensile deformation behavior at a high temperature of 650 °C of the GH4706 Ni-based superalloy after stabilization treatment. Transmission electron microscopy (TEM) and secondary ion mass spectrometry (SIMS) were employed to characterize microstructural evolution and elemental redistribution to clarify the microscopic mechanisms by which stabilization treatment enhanced the high-temperature ductility of the GH4706 alloy. The experimental results indicated that better high-temperature plasticity was obtained, although tensile strength decreased slightly after stabilization. This improvement was mainly attributed to the precipitation of the η phase (Ni_3_Ti) and its synergistic interaction with the matrix, which effectively enhanced the plastic deformation capacity of the GH4706 alloy at elevated temperatures. Moreover, η phase precipitation and elemental segregation enhanced grain boundary stability, thus inhibiting crack initiation and delaying necking. SIMS analysis revealed that boron, phosphorus, and sulfur showed significant segregation along grain boundaries during 650 °C tensile testing following stabilization—an effect considered crucial to the observed ductility enhancement. TEM observations further indicated that the interaction between η phase precipitation and the nucleation and evolution of stacking faults during deformation together reduced local stress concentrations and promoted uniform plastic deformation.

## 1. Introduction

Nickel-based superalloys are the primary choice for aero-engine hot-section components, such as turbine blades, combustion chambers, and turbine disks, owing to their exceptional high-temperature strength, creep resistance, and phase stability [1,2,3,4]. GH4706 alloy is derived from GH4169 alloy by the removal of Mo, reduction in Nb, Al, Cr, and Ni, and the addition of Ti and Fe [5,6,7]. Compared with GH4169 alloy, GH4706 alloy has the advantages of low cost, low segregation, excellent machinability and superior workability, making it well suited for gas-turbine components and large turbine-disk forgings operating below 700 °C [8,9].

The high-temperature mechanical performance of such alloys is strongly governed by their microstructural evolution. In many nickel-based systems, the γ′ precipitate (Ni_3_(Al, Ti)) acts as the principal strengthening phase, with its size, distribution, and volume fraction directly correlated with strength and creep resistance [10,11,12,13,14]. In addition, previous studies have addressed η phase precipitation (Ni_3_Ti) and its effect on the mechanical properties of various nickel-based superalloys. Depending on its morphology and distribution, η phase precipitation can enhance grain boundary stability or, conversely, degrade matrix strength by depleting γ′-forming elements [15,16,17,18,19,20]. In GH4706, stabilization treatments have been shown to induce significant η phase formation at grain boundaries, accompanied by compositional changes in the γ matrix [21,22,23]. Thus, controlling η phase precipitation is critical to tailoring the strength–ductility trade-off at high temperatures.

Grain boundary chemistry further modulates high-temperature behavior. Beneficial trace elements such as boron and phosphorus have been reported to enhance grain boundary cohesion, resist crack initiation, and improve creep/fatigue life in alloys like IN718, GH761, and HT700 [24,25,26,27,28,29]. Conversely, sulfur generally promotes grain boundary embrittlement by forming deleterious sulfides [30]. While these segregation phenomena are known, their interaction with precipitate evolution and defect mechanics during high-temperature deformation in GH4706 remains poorly understood.

More importantly, despite extensive studies on the γ′ and η phases, there has been limited in-depth analysis of how η phase precipitation interacts with stacking faults, deformation twins, and dislocation motion at the η/γ interface during service-relevant tensile deformation. This microstructural mechanical coupling is particularly relevant for GH4706 bar stock used in turbine-disk manufacturing: improved ductility at the bar stage mitigates cracking and strain localization during subsequent die forging.

In this work, we investigate forged GH4706 bar stock subjected to stabilization treatment at 830 °C for 3 h, followed by tensile testing at 650 °C. Using SIMS and TEM, we examine the coordinated evolution of elemental segregation, η phase precipitates, γ′ phase changes, and grain boundary, and analyze their interplay with dislocations, stacking faults, and twins. The study seeks to clarify the coupled mechanisms that enhance high-temperature ductility, providing both theoretical insights and practical guidance for heat treatment optimization in aero-engine turbine-disk production.

## 2. Experimental Procedure

The GH4706 alloy used in this study has the chemical composition listed in Table 1 [31].

**Figure 1:**
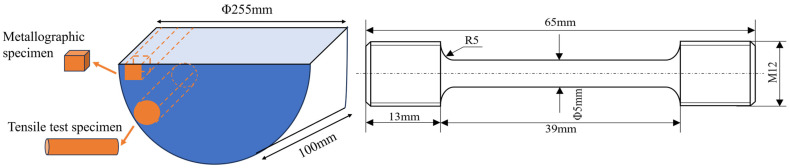
Schematic diagrams of specimen extraction location for GH4706 bars and the tensile specimen.

GH4706 bar stock was selected as the experimental material. Stabilization treatment was carried out at 830 °C for 3 h, followed by water quenching to room temperature. Both untreated and stabilized bars were machined into tensile specimens as shown in Figure 1. To ensure the reliability and repeatability of mechanical property data, one parallel tensile specimen was prepared for each test condition. Uniaxial tensile tests were performed on an Instron 5982 machine (Instron, Norwood, MA, USA) at room temperature (25 °C) and at 650 °C in accordance with GB/T 228.1-2010 [32] and GB/T 228.2-2015 [33]. Tensile strength, yield strength, and elongation were recorded for each specimen, and the values analyzed represent the average of the two tests for each condition.

Six parallelepiped specimens (12 mm × 10 mm × 10 mm) were cut from the edge region of the GH4706 bar. Three specimens were stabilization treated at 830 °C for 3 h and then water-quenched, while the remaining three served as untreated controls. The metallographic preparation of these specimens followed the standardized workflow: all specimens were first ground sequentially with 400#, 800#, 1200#, and 2000# silicon carbide sandpapers, with the specimen rotated 90° each time the sandpaper was replaced and cold water cooling applied during grinding; subsequent two-step polishing involved 30 min of polishing with 3 μm diamond polishing fluid and 15 min of polishing with 0.04 μm colloidal silica polishing fluid to achieve a mirror-like surface. The polished specimens were corroded at room temperature for 30 s in a solution of 20 g CuCl_2_ + 100 mL HCl + 100 mL C_2_H_5_OH, with gentle shaking during corrosion to ensure uniformity. After corrosion, the specimens were rinsed with deionized water, dehydrated with absolute ethanol, and dried with cold compressed air. Microstructural observations were then carried out using a Gemini SEM 300 field-emission scanning electron microscope (SEM) (Carl Zeiss Microscopy GmbH, Oberkochen, Germany), and the γ′ precipitate size was quantified using Image-Pro Plus 6.0 software.

Prior to fracture-surface examination, tensile-failed specimens were immersed in acetone and ultrasonically cleaned for 15 min. The fracture morphologies were then observed under a SIGMA 300 field-emission SEM (Carl Zeiss Microscopy GmbH, Oberkochen, Germany) at an accelerating voltage of 10 kV.

Grain boundary segregation of elements was analyzed by time-of-flight secondary ion mass spectrometry (TOF-SIMS 5-100) (IONTOF GmbH, Münster, Germany) on metallographic samples taken from stabilization-treated specimens after room-temperature and high-temperature tensile tests. Before analysis, samples were pre-sputtered with a Cs^+^ source (1 kV, 2 nA) for 120 s over a 300 μm × 300 μm area to a depth of ~30 nm. Fast-imaging mode used Bi_3_^++^ at 30 kV to map segregation over 50 μm × 50 μm at 256 pixel × 256 pixel resolution.

Transmission electron microscopy (TEM) observations were performed on a JEM-F200 (JEOL Ltd., Tokyo, Japan) at 200 kV. Fracture-edge specimens were sectioned into 8 mm × 0.5 mm slices. TEM foils were prepared by grinding to ~50 μm, punching into 3 mm disks, then electrolytically thinning at −20 °C and 24 V in a solution of 90% ethanol (C_2_H_5_OH)  +  10% perchloric acid (HClO_4_) using a Struers TenuPol-5 until perforation. Selected-area electron diffraction (SAED), high-resolution TEM (HRTEM), high-angle annular dark-field STEM (HAADF-STEM), and EDS line scans were employed to characterize precipitate morphology, dislocation configurations, phase identities, and elemental distributions.

## 3. Results and Discussion

### 3.1. Microstructure Characterization

**Figure 2:**
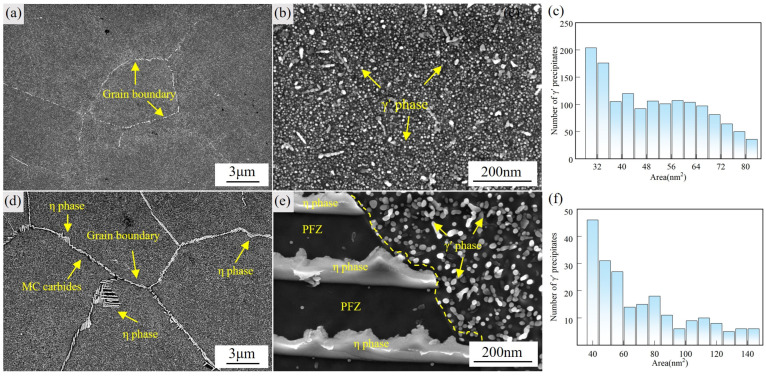
Microstructures of specimens before and after stabilization treatment. (**a**) SEM image of grain boundary morphology before stabilization treatment. (**b**) SEM image of γ′ precipitates. (**c**) Area distribution histogram of γ′ precipitates. (**d**) SEM image of grain boundary morphology after stabilization treatment. (**e**) SEM image of η phase precipitates in the PFZ and γ′ precipitates within and adjacent to the PFZ. (**f**) Area distribution histogram of γ′ precipitates.

Figure 2 illustrates the morphology and spatial distribution of η phase and γ′ precipitates in GH4706 alloy before and after stabilization treatment. SEM micrographs of the as-forged and stabilized samples are shown in Figure 2a,d, respectively. In Figure 2a, γ′ precipitates are uniformly dispersed throughout the matrix of the as-forged specimen. Figure 2b highlights fine γ′ particles and their homogeneous distribution. The particle-size histogram in Figure 2c shows that the number density of γ′ precipitates decreases with increasing particle area, with most particles occupying the 32–85 nm^2^ range. After stabilization at 830 °C for 3 h (Figure 2d), rod- and needle-shaped η phase precipitates are observed along grain boundaries, accompanied by the formation of precipitate-free zones (PFZs). Figure 2e reveals the coexistence of η phase and γ′ phase and clearly delineates the PFZ, which arises because titanium (Ti) and niobium(Nb)-rich η phase precipitates deplete these elements from adjacent regions, thereby suppressing γ′ nucleation [34,35]. The γ′ size distribution for the stabilized alloy (Figure 2f) shows a marked reduction in precipitate number due to Ti consumption by the η phase, inhibiting γ′ nucleation, with the average area increasing from 57 nm^2^ to 79 nm^2^.

### 3.2. Tensile Properties

**Figure 3:**
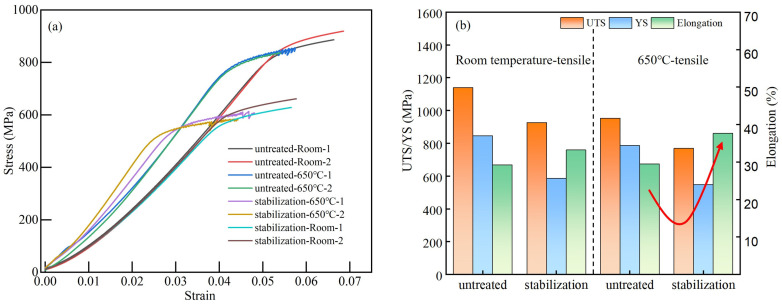
(**a**) Stress–strain curve; (**b**) tensile properties of GH4706 alloys tested at different temperatures.

Figure 3 compares the mechanical properties of GH4706 alloy under room-temperature tensile testing and 650 °C high-temperature tensile testing conditions before and after stabilization treatment. In room-temperature tests, the as-forged specimen has a tensile strength of 1141.5 MPa and a yield strength of 846.5 MPa, but only 29.25% elongation. After stabilization treatment, the tensile strength and yield strength decrease to 927 MPa and 586.5 MPa, while elongation increases to 33.25%. At the temperature of 650 °C, the as-forged sample has a tensile strength of 954 MPa and a yield strength of 787.5 MPa with an elongation of 29.5%. After stabilization treatment, the tensile strength and yield strength further decrease to 771 MPa and 549.5 MPa, whereas elongation increases significantly to 37.75%.

**Figure 4:**
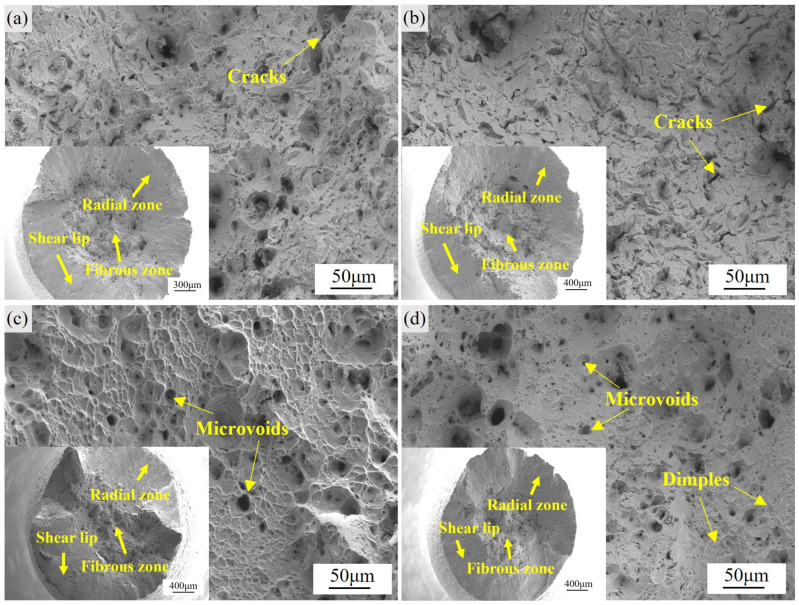
Fracture morphology of GH4706 alloy specimens after tensile testing: (**a**) Microscopic fracture surface of specimens at room temperature before stabilization treatment. (**b**) Microscopic fracture surface of specimens tested at a temperature of 650 °C before stabilization treatment. (**c**) Microscopic fracture surface of specimens at room temperature after stabilization treatment. (**d**) Microscopic fracture surface of specimens tested at a temperature of 650 °C after stabilization treatment.

Figure 4 shows the fracture morphologies of GH4706 alloy after tensile testing at room temperature and 650 °C, where Figure 4a–d are microscopic fracture surfaces with corresponding macroscopic fractures in the lower left. At room temperature, the untreated specimen (Figure 4a) has a narrow shear lip, with sparse small microvoids and a few flattened tear facets in radial and fiber zones, indicating mixed brittle-ductile fracture. In contrast, the stabilized specimen (Figure 4c) exhibits a notably broadened shear lip and dense, large, deep dimples in the fiber zone—reflecting extensive microvoid nucleation, coalescence and growth, consistent with reduced strength and increased elongation.

At 650 °C (Figure 4b,d), both specimens form wider shear lips and more prominent fiber zones. However, the stabilized alloy (Figure 4d) has larger, coalesced dimples with smooth, uniform void boundaries (no brittle features), while the untreated sample (Figure 4b) contains isolated microcrack networks between smaller, dispersed dimples. Overall, stabilization treatment promotes grain boundary η phase precipitation and precipitate-free zones, enhancing microvoid mechanisms. The fracture surfaces clearly demonstrate the coupled effect of improved ductility and reduced strength.

### 3.3. Influence of Elemental Segregation

**Figure 5:**
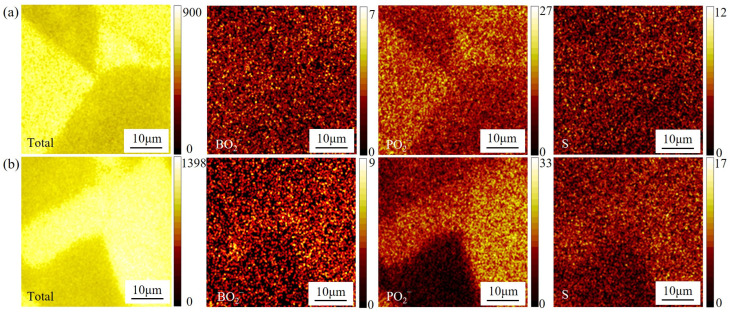
SIMS images showing elemental segregation in GH4706 alloy specimens: (**a**) specimens before stabilization treatment; (**b**) specimens after stabilization treatment.

Figure 5 shows the SIMS mapping of grain boundary segregation in the GH4706 alloy before and after stabilization treatment tested at a temperature of 650 °C, revealing clear differences in the distribution of sulfur (S), boron (B), and phosphorus (P) and their cooperative effects on high-temperature ductility. In Figure 5a, the three elements show only slight enrichment at grain boundaries, with broadly dispersed signals. By contrast, S, B, and P signals are markedly intensified and localized along specific boundaries in Figure 5b, showing pronounced and localized enrichment.

Elevated temperature can accelerate atomic diffusion, driving solute migration toward high-energy sites such as grain boundaries. This behavior corresponds to the expanded enrichment zones observed after high-temperature deformation, because B and S compete for adsorption at the boundary. B preferentially occupies active sites and forms stable borides or segregates in elemental form, thereby reinforcing boundary cohesion and mitigating deleterious S segregation, because of its smaller atomic radius and higher interfacial binding energy [36]. Meanwhile, P and its oxides (e.g., PO_2_^−^) fill grain boundary vacancies and pin dislocation motion, further stabilizing the boundary structure.

Notably, Ni–S sulfides (e.g., Ni_3_S_2_) approach their melting point (~797 °C) at the temperature of 650 °C and adopt a semi-molten state, which modestly reduces boundary strength but promotes grain boundary sliding, thus enhancing high-temperature deformation coordination [37]. In this semi-molten condition, the rigid metallic and metal–sulfur bonds within Ni_3_S_2_ are partially replaced by liquid-phase viscous interactions, which lack crystalline lattice support and therefore interrupt direct atomic bonding between adjacent grains, reducing intrinsic grain boundary cohesion. Upon cooling, the re-solidified Ni_3_S_2_ may form thin, brittle films along boundaries, further lowering their load-bearing capability.

Concurrently, the synergistic effect of B and P segregation suppresses crack initiation and propagation during sliding, effectively counteracting the detrimental influence of such cohesion loss. As a result, enriched regions optimize atomic bonding at the boundary, making the alloy more prone to ductile grain boundary accommodation rather than brittle fracture under high-temperature load. This mechanism is consistent with the significant elongation improvement observed at 650 °C (Figure 3) and the more uniform distribution of dimples in the post-fracture morphology (Figure 4d), collectively explaining the intrinsic link between multiple elemental microsegregations and enhanced macroscopic plasticity.

### 3.4. Evolution of Microstructure and Its Influence on High-Temperature Tensile Properties

**Figure 6:**
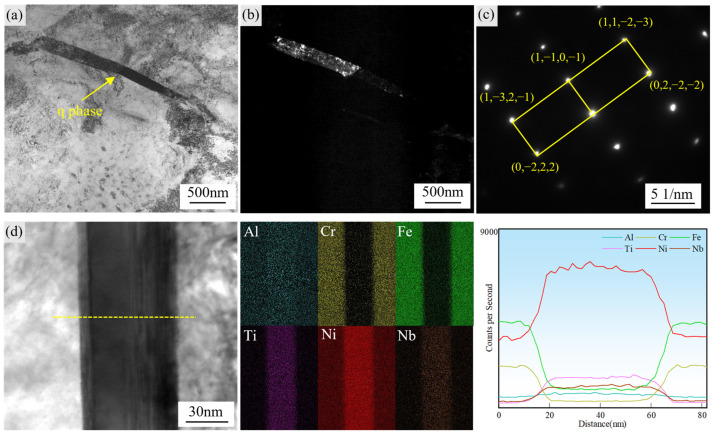
Microstructural and compositional characterization of the η phase in GH4706 alloy after stabilization treatment: (**a**) Bright-field TEM image of the η phase. (**b**) Dark-field TEM image of the η phase. (**c**) Selected area electron diffraction (SAED) pattern of the η phase. (**d**) HAADF image of the η phase and elemental mapping along the yellow dashed line, showing the elemental distribution of Al, Cr, Fe, Ti, Ni, and Nb in the η phase.

Figure 6 displays high-resolution bright-field TEM images of η phase precipitates in GH4706 alloy after stabilization treatment. At this temperature, η phase nucleation occurs preferentially along grain boundaries due to their inherently high interfacial energy. The formation of the η phase at these sites significantly reduces the total free energy of the system, providing the primary thermodynamic driving force for precipitation [38,39]. In addition, the elevated defect density at grain boundaries accelerates atomic diffusion, while Ti segregation in these regions further facilitates η phase nucleation and subsequent growth [40]. According to Hou et al. [15], there exists a specific orientation relationship between the {0001} planes of η phase and the {111} planes of the γ matrix; this crystallographic pairing minimizes interfacial energy and favors coherent growth along the {0001}η//{111}γ direction, driving grain boundary migration. In the thickness direction of growth, however, the η/γ interface is incoherent, leading to the characteristic lath morphology observed in Figure 2d.

Figure 6d presents an HAADF-STEM image of η phase precipitates and the corresponding TEM-EDS line scan. Both Ti and Ni signal intensities increase simultaneously within the η phase laths with a Ti/Ni peak ratio of approximately 1:3, consistent with the stoichiometry of Ni_3_Ti. Surrounding the η phase plates, the γ matrix shows Ti depletion, confirming significant elemental migration and redistribution during η phase precipitation.

Dislocations, a primary type of microstructural defect in alloys, exhibit mobility that is governed by multiple factors. Previous studies have demonstrated that the precipitation of the η phase along grain boundary regions during heat treatment plays a decisive role in controlling both the slip behavior of dislocations and their spatial distribution at grain boundaries, a mechanism that largely determines the alloy’s high-temperature tensile properties. Consequently, it is essential to examine how the η phase influences dislocation motion.

**Figure 7:**
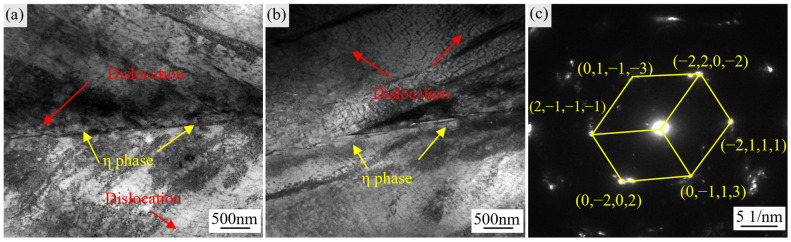
TEM images of η phase and dislocations in GH4706 alloy after 650 °C tensile deformation: (**a**,**b**) Bright-field TEM images showing the morphologies of η phase and dislocations at grain boundaries. (**c**) Selected area electron diffraction (SAED) pattern of η phase at grain boundaries.

Figure 7 presents TEM observations of η phase precipitates located at grain boundaries, together with surrounding dislocations. Plate-like η phase precipitates form continuously along the boundary, creating an effective pinning effect on gliding dislocations. This pinning action restricts early dislocation pile-up, suppresses microcrack nucleation at the grain boundary, and consequently improves boundary strength. The TEM image in Figure 7b shows that dislocation networks are concentrated near grain boundaries and η phase plates, indicating that the η/γ interfaces act as strong barriers to dislocation motion. These barriers limit local dislocation interactions and pile-ups, thereby reducing the tendency for premature microcrack initiation and delaying crack propagation.

Such stable dislocation configurations not only inhibit crack initiation but also delay the evolution of microdamage, manifesting macroscopically as enhanced plastic deformation capability. As shown by the high-temperature tensile test results in Figure 3, the elongation of the alloy after stabilization treatment is significantly improved; this improvement results from the interfacial interactions between η phase precipitates and dislocations at the microscale, which mitigate strain localization and provide additional cooperative slip pathways [7], ultimately enhancing the overall plasticity at the temperature of 650 °C.

**Figure 8:**
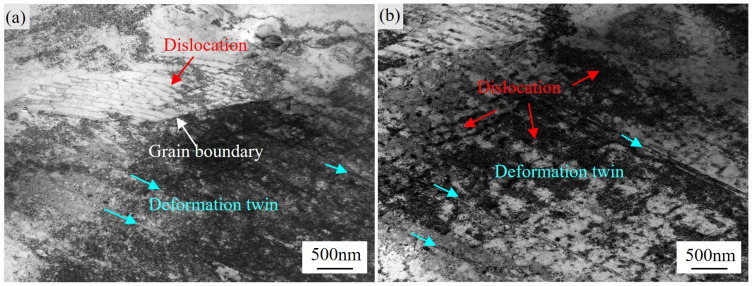
Dislocation and deformation twin characteristics in GH4706 alloy: (**a**) Bright-field TEM image of dislocations, grain boundary, and deformation twins. (**b**) Bright-field TEM image of the distribution of dislocations and deformation twins.

During high-temperature tensile deformation at 650 °C, a high density of dislocations in the GH4706 alloy exhibits significant mobility within grain interiors. These dislocations interact and entangle at local stress concentration sites, forming dense networks. As shown in the TEM micrographs of Figure 8a,b, portions of these dislocations form a grid-like arrangement (indicated by the red arrows), signifying that they have undergone dynamic rearrangement and stabilization under elevated temperature. With increasing strain, the accumulated stress gradients during dislocation slip trigger the nucleation of deformation twins in favorably oriented grains (highlighted by the cyan arrows). These twins interact with adjacent slip systems, effectively reducing local strain localization and enhancing the ability to accommodate plastic deformation, which is an effect analogous to the TWIP (twinning-induced plasticity) mechanism observed in high-Ni alloys [41]. Moreover, deformation twins create additional slip pathways for dislocation motion, promoting intergranular strain transfer. This synergistic “dislocation–twin” deformation mechanism both delays the onset of microcrack formation due to stress concentration and enhances overall grain-scale deformability, thereby providing a microstructural explanation for the experimentally observed increase in elongation at high temperature.

At 650 °C, the activation of twinning in GH4706 can be rationalized by its relatively low stacking fault energy, which facilitates the extension of partial dislocations and the accumulation of stacking faults. When dislocations pile up at grain boundaries or at precipitate/matrix interfaces, the resulting local stress concentration provides favorable conditions for twin nucleation. Although dislocation climb and cross-slip are more active at this temperature, conventional slip alone may not be sufficient to accommodate the imposed plastic strain, and twinning thus acts as an auxiliary mechanism to relieve local stress. In contrast, no equiaxed recrystallized grains, necklace structures, or high-angle boundaries that are typical of dynamic recrystallization were observed in TEM images, suggesting that twinning rather than DRX is the dominant deformation mechanism under the present testing conditions.

**Figure 9:**
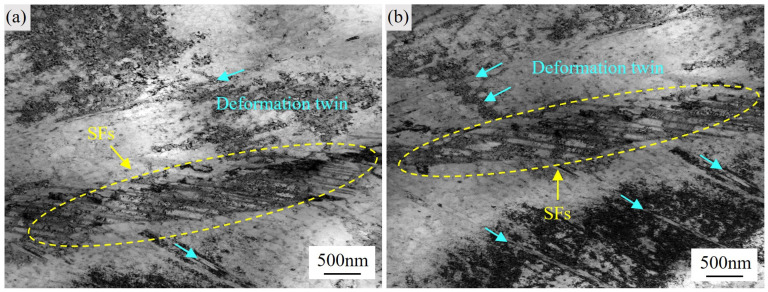
Microstructural characteristics of stacking faults (SFs) and deformation twins in GH4706 alloy: (**a**,**b**) Bright-field TEM images showing the distribution of stacking faults and deformation twins.

In the γ matrix interior, numerous parallel stacking faults (SFs), indicated by yellow dashed loops in Figure 9a,b, form as a consequence of dislocation activation and subsequent slip. These planar fault structures not only act as glide barriers directing dislocation motion but also promote SF thickening and bundling and locally induce the nucleation and propagation of deformation twins, indicated by cyan arrows.

Stacking faults are lattice defects arising from disruptions in the stacking sequence; by modifying the local stress field, they impede dislocation glide and increase yield strength, while also mediating the distribution of plastic strain during high-temperature deformation. As strain accumulates further, SFs progressively evolve into twin embryos, which nucleate and grow along mirror-symmetric interfaces through interfacial softening, thereby alleviating localized stress concentrations.

Although deformation twinning is typically more pronounced at low temperatures, in the present study twins are nevertheless observed under 650 °C tensile conditions, a behavior closely linked to the suppression of cross-slip. Cross-slip, as an important dislocation relaxation mechanism in FCC metals that is usually activated at elevated temperatures to release internal stress, is restricted here by low stacking-fault energy (SFE) and the high local strain-rate environment of the test. Literature reports note that cross-slip depends on the constriction of extended screws. When this process is hindered, cross-slip is suppressed and twinning becomes the dominant alternative [42]. Deformation twins thus not only relieve internal stresses effectively but also enhance local strain compatibility, leading to improved overall ductility. Through this synergistic “stacking-fault–twin” mechanism, the GH4706 alloy exhibits enhanced deformation versatility at high-temperature. The inhibition of cross-slip permits twinning to operate even at the temperature of 650 °C, thereby contributing to superior high-temperature elongation and overall plasticity.

**Figure 10:**
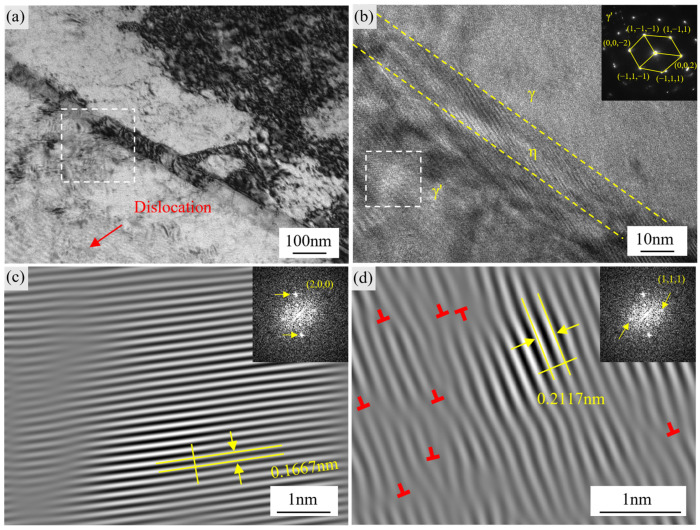
Characteristics of η phase, dislocations, and stacking faults in GH4706 alloy after 650 °C tensile deformation: (**a**) Bright-field TEM image of η phase; (**b**) HRTEM image at the phase boundary; (**c**,**d**) FFT images corresponding to the marked regions in (**a**).

Because of the semi-coherent relationship between the η phase and the γ matrix, dislocations tend to accumulate at the interface [43]. During high-temperature tensile deformation at 650 °C, the alloy’s excellent plasticity is attributed to the interactions between dislocations and the interfaces of the η phase, γ′ phase, and γ matrix in its multiphase microstructure, which form a composite defect network. At the γ′–η phase interface, the directional migration of Ti atoms leads to a significant chemical potential gradient, which causes local tensile and compressive deformations at the sub-angstrom scale in the lattice near the interface, forming activation sources for dislocations in regions of shear stress concentration. When the applied tensile stress exceeds the critical resolved shear stress and overlaps with the interface shear field, dislocations first slip along the (111) crystal plane [44], as shown in Figure 10d, where the interplanar spacing is measured at approximately 0.21 nm. IFFT reconstruction further reveals dislocation bundles with intermittent strip-like interruptions—a signature of accumulated lattice strain.

As tensile strain accumulates further, dislocations from the same or adjacent (111) crystal planes cross-slip and intertwine to form a multidirectional dislocation network. Local strain energy accumulates at the network nodes, reaching the twin nucleation threshold and leading to the formation of mirror-symmetric twin bands of only a few nanometers in width, as shown in Figure 9a,b. As strain continues to accumulate, these initial dislocations cross-slip across different crystal planes, gradually building a multidirectional dislocation network. The IFFT in Figure 10d clearly shows that lattice fringes are grouped on the same or adjacent crystal planes and exhibit characteristic interruptions, indicating that local stress accumulation continues to activate the repeated nucleation and sliding behavior of the lattice. This dislocation network has two effects: on one hand, it offers “high-speed channels” for subsequent dislocations, enabling rapid dislocation proliferation; on the other hand, strain energy accumulation at the cross-slip nodes reduces the twin nucleation barrier, leading to the extension of mirror-symmetric deformation twin bands along the (111) plane.

In Figure 10c, the lattice fringes are clearly visible with a spacing of approximately 0.1667 nm, with slight bending of the fringes and local displacement, suggesting dislocation slip behavior within the lattice, potentially inducing lattice shear distortions. In Figure 10d, the lattice fringe spacing is 0.2117 nm, displaying more pronounced fringe deflections and local inhomogeneities, reflecting enhanced lattice distortion due to high-temperature tensile stress. These fringe features are due to the misalignment of crystal planes caused by dislocation slip within the lattice, indicating that at 650 °C, the plasticity of the GH4706 alloy is primarily achieved through the cooperative motion of dislocations on different slip systems. During stretching, elevated temperature not only reduces the energy barrier for dislocation motion but also increases the spatial extent of dislocation slip, making the plastic deformation process more thorough and uniform.

## 4. Conclusions

(1) Stabilization treatment significantly enhances the high-temperature ductility of the GH4706 superalloy at 650 °C by promoting uniform deformation and delaying necking. Compared with the untreated samples, the elongation of treated samples tested at a temperature of 650 °C is markedly increased.

(2) SIMS measurements show that, during tensile testing at 650 °C, B, P, and S segregate substantially to the grain boundaries in the stabilized GH4706 alloy. The formation of high-melting-point compounds by B and S reduces their detrimental impact, while P enhances boundary stability; their interaction with the η/γ interfaces optimizes grain boundary ductility and cooperatively elevates the alloy’s high-temperature plasticity.

(3) TEM analysis reveals that the η/γ interfaces formed after stabilization treatment act as barriers to dislocation motion, leading to the accumulation of partial dislocations and the formation of stacking faults nearby. During continued straining, these stacking faults can transform into deformation twins, which, together with dislocation networks, help to disperse local stress concentrations and provide additional slip systems. This “stacking fault–twin” mechanism significantly improves strain accommodation and contributes to the enhanced high-temperature ductility of the GH4706 alloy.

## Figures and Tables

**Table 1:** Chemical compositions of GH4706 alloy (mass fraction) [31].

Elements	C	B	P	Ti	Al	Nb	Cr	Fe	Ni
wt%	0.01	0.003	0.007	1.66	0.29	2.92	16.1	37.1	Bal.

## Data Availability

The original contributions presented in this study are included in the article. Further inquiries can be directed to the corresponding author.
